# The effect of N-acetyl cysteine injection on renal function after coronary artery bypass graft surgery: a randomized double blind clinical trial

**DOI:** 10.1186/s13019-021-01550-7

**Published:** 2021-06-05

**Authors:** Fatemeh Javaherforooshzadeh, Zahra Shaker, Mahboobeh Rashidi, Reza Akhondzadeh, Fatemeh Hayati

**Affiliations:** 1 Department of anesthesia, Ahvaz Anesthesiology and Pain Research Centre, Ahvaz Jundishapur University of Medical Sciences, Ahvaz, Iran; 2 Department of Internal Medicine, School of Medicine. Chronic Renal Failure Research Center, Ahvaz Jundishapur University of Medical Sciences, Ahvaz, Iran

**Keywords:** N-acetyl-cysteine, Acute kidney injury, Coronary artery bypass graft surgery

## Abstract

**Background:**

This study aimed to compare the effects of N-acetyl cysteine on renal function after coronary artery bypass graft surgery.

**Methods:**

In this randomized clinical trial conducted in Golestan Hospital, Ahvaz, Iran, 60 candidates for coronary artery bypass graft surgery were selected and divided into two N-acetyl cysteine and control groups (30 people each). Patients received 3 (2 intraoperative and 1 postoperative) doses of IV N-acetyl cysteine (100 mg/kg) (*n* = 30) or placebo (*n* = 30) over 24 h. Prescription times were as follows: after induction of anesthesia, in the Next 4 h, and in the 16 h after on. Primary outcomes were serum levels of BUN and Cr, at baseline,4 and 48 h after surgery**.** And also need renal replacement therapy (RRT). Secondary outcomes included the hemodynamic variables, Blood products transfusion.

**Results:**

There were significant differences in BUN between groups at 4 h (*P* = 0.02) and 48 h after surgery (*P* = 0.001) There were significant differences in Cr level between groups at 4 h (*P* < 0.001) and 48 h after surgery (P = 0.001).

MAP at different times (at 4 h *p* = 0.002 and 48 h after surgery P < 0.001) were significantly different between the two groups. There was a significant difference between the two groups in terms of the unit of Packed cell transfusion (*P* = 0.002) and FFP transfusion (*P* < 0.001).

**Conclusion:**

In the present study, we found that administration of N-acetyl cysteine can reduce the incidence of acute kidney injury in patients undergoing coronary artery bypass graft surgery and improved kidney functions.

**Trial registry:**

IRCT20190506043492N3 **Registered at** 2020.06.07.

## Background

Open heart surgery for coronary artery bypass graft (CABG) is one of the major surgeries in adults [1]. Kidney failure is one of the most common complications of coronary artery bypass graft surgery. Even a very low increase in serum creatinine levels in these patients has been associated with an increase in mortality [2]. Acute renal failure after coronary artery bypass graft (CABG) increases mortality, morbidity and the length of hospitalization in intensive care unit and some cases causes dialysis [2, 3].

The main factors leading to acute renal failure after CABG are pre-operation renal dysfunction, heart failure, Diabetes Mellitus. Among other risk factors such as age, cross-clamp time, type of operation, poisons, metabolic factor, ischemia and reperfusion injury, activation of neurohormonal factors, inflammation, and oxidative stress responses, duration of CPB time is considered as an independent factor which increases the risk of post-CABG acute renal failure [4].

The frequency of acute renal failure necessitating renal replacement therapy (RRT) subsequent CPB is assessed to occur in up to 3.7% of patients, whereas azotemia, not requiring RRT, occurs in about 11.4% of patients [5].

Early diagnosis of Acute Kidney Injury (AKI) is very important for effective prevention and treatment. Effective AKI treatment is depending on early monitoring of biomarkers. Serum creatinine levels are currently used to diagnose renal failure. However, this is not a reliable index during acute changes in kidney function [6], because its rate can be in the normal range even in patients with kidney damage greater than 50% [6]. Renal failure is poor kidney function, which may be due to decreased blood flow to the kidneys caused by kidney artery disease. In general, the kidneys regulate fluid and electrolytes and blood pressure, however, proper kidney function may be impaired in a condition called renal artery stenosis, in which the feeding arteries of the kidneys are narrowed, restricted, and weakened [7].

There are several measures, including maintaining sufficient intravascular volume, adequate perfusion, and avoiding nephrotoxic drugs to prevent AKI. Some drugs such as Calcium Channel Blockers and Statins are also used to prevent the incidence of AKI [8]. To achieve this goal, many researchers examined antioxidant molecules, including N-acetyl cysteine.

N-acetyl cysteine is an antioxidant derived from the amino acid cysteine is one of the medicines used to attenuate ischemic renal failure through nitric oxide–independent arteriolar vasodilation while functioning as an antioxidant and it can be preventing nephropathy caused by contrast agents are among the indications [9]. It only shows antioxidant and anti-inflammatory effects at high doses. It seems the application of N-acetyl cysteine supplementation increases Glutathione, which is the main antioxidant in the body. Also, it reduces the formation of inflammatory cytokines such as interleukin-8 and tumor necrosis factor-alpha [9] N-acetyl cysteine is the acetyl form of L-cysteine, which is converted into metabolites in the body that can synthesize glutathione, promote detoxification, and also has a direct effect on removing free radicals. It is used in various therapeutic cases such as acetaminophen poisoning, bronchitis, drug addiction, and schizophrenia. This compound is a derivative of cysteine in which an acetyl group is attached to the amino group in cysteine. NAC is essentially a precursor which is converted into cysteine (in the intestine by aminoacylase-1) and is absorbed into the bloodstream [10]. Cysteine is a key component of glutathione and therefore, administration of NAC fills glutathione reserve. NAC can also be used as a general antioxidant that helps to reduce symptoms of many diseases that have been worsened by reactive oxygen species (ROS). Researches show that the use of N-acetyl cysteine can prevent acute renal failure in patients with renal failure [11].

Since no effective prevention methods have been found to inhibit acute renal failure after CABG surgery, this study is aimed to investigate the effect of N-acetyl cysteine injection on renal function after coronary artery bypass graft surgery.

## Material and method

### Study design

This double-blind randomized clinical trial study was conducted in Golestan Hospital, Ahvaz, Iran, from Agust 2020 to January 2021. Sixty patients undergoing elective on-pump coronary artery bypass graft surgery were included.

#### Ethical statement

This paper was part of a thesis Ethics code: (IR.AJUMS.REC.1398.392 was received from Anesthesiology and Pain Research Center, Ahvaz Jundishapur University of Medical Sciences, Ahvaz, Iran. The RCT code of this study wasIRCT20190506043492N3.The Grant no of this study was PAIN-9817.

After clearly explaining the objective and potential risks and benefits of the study, a written consent form for participation in the study was obtained from all patients.

### Setting and patients

#### Inclusion criteria included

Candidates for elective open CABG, aged 30 to 70 years.

#### Exclusion criteria included

The patient is not satisfied to participate in the study, renal and dysfunction based on KDIGO criteria [12], history of steroids and anti-inflammatory taking, acute myocardial infarction within fewer than 1 weeks, redo surgery, ejection fraction less than 30%, and complex surgeries.

#### Randomization and blindness

The patients were randomized 1:1 to receive either (N-acetyl cysteine) or normal saline as control. To ensure that, the patients the surgeon and the investigators were blind to the treatment group before the study begins, we used a computer-generated allocation-concealment process before recruiting the patients. The patients, surgeon, and investigator were unaware of the type of injectable drug and the surgeries performed by the same surgeon (The injectable drug was prepared by the researcher and named 1 and 2).

### Sample size

The sample size of this study was calculated using the sample size estimation formula. The 95% confidence interval (CI) level was considered. The study population consisted of 60 patients. Based on the previous data [13].

$$ \mathrm{n}=\frac{\mathrm{Z}1\hbox{-} \upalpha /22.\kern0.5em \mathrm{P}\left(1\hbox{-} \mathrm{P}\right)}{\mathrm{d}2} $$

After entrance to the operating room, standard monitoring included five-lead electrocardiography, pulse oximetry, and arterial line for continuous blood pressure monitoring, and blood gases were inserted. The anesthesia technique, the surgeon, and the cardiopulmonary bypass procedure was intended to be as similar as possible. The patients after surgery were admitted to the cardiovascular ICU. Protocol for sedation (Dexmedetomidine 0/1–1 μg /kg/min), and management of mechanical ventilation (SIMV mode of ventilation) were similar for all patients and if they matched the weaning criteria, were extubated.

### Intervention

In the intervention group, after induction of anesthesia and intubation, and hemodynamic stabilization, 100 mg/kg N-acetyl cysteine that diluted in 100 ccs normal saline will be administered intravenously in 20 min, 100 mg/kg in the Next 4 h and100 mg/kg in the 16 h after on. In the control group, normal saline will be administered in the same volume and at the same times.

**Primary outcomes** were serum levels of BUN and Cr, at baseline,4 and 48 h after surgery**.** And also the need for renal replacement therapy (RRT).

**Secondary outcomes**: Included the hemodynamic variables, the number of unit of postoperative Paced red cell and FFP transfusion (Paced cell transfusion was performed if the hemoglobin was less than 7 mg/dl and FFP transfusion were performed if oozing was present and one FFP unit per blood unit), Urine output.

### Statistical analysis

All statistical analysis was completed using SPSS version 22. Descriptive statistics are reported as mean ± standard deviation for continuous variables and as frequency and percentage for categorical variables. Parameters with a normal distribution were compared using the unpaired t-test and repeated measurement, Statistical significance was defined as a *p*-value < 0.05**.**

## Results

**Fig. 1:**
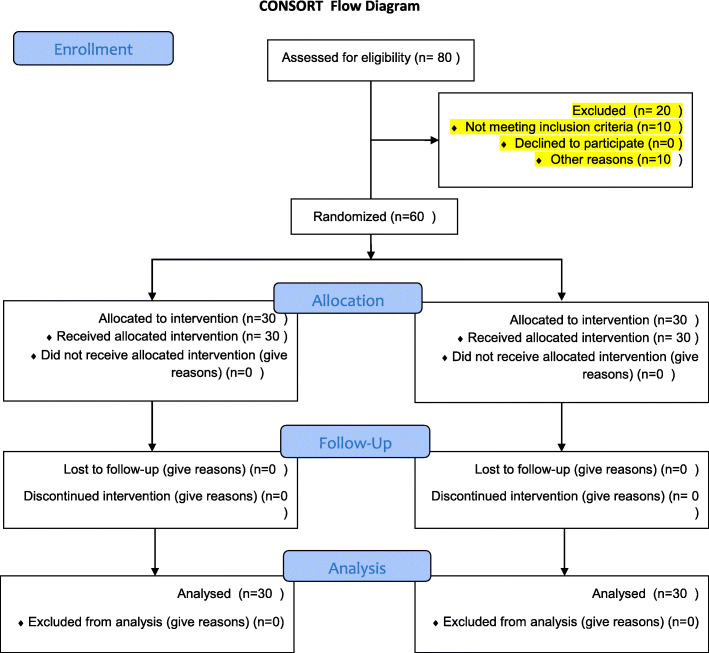
Eighty subjects were enrolled in the study, of which 60 met the criteria for the study and consented to participate. Participants were divided into two groups (each with 30 subjects)

During the study period from August 2020 to January 2021, 110 patients undergoing elective CABG surgery were qualified to contribute to the trial. Eighty patients agreed to contribute with written informed consent. Among them, 10 patients did not have inclusion criteria, 5 patients had a history of renal disease, and 5 patients were given steroid drugs. Finally, 60 patients were registered in the study and were allocated into two groups of N-acetyl cysteine and control (normal saline), 30 patients each **(**Fig. 1**).**

There were no significant differences between the two groups in terms of demographic characteristics (*P* > 0.05) (Table 1).

**Table 1:** Demographic Data of patients in N-acetyl cysteine group and control group

Parameter	N-acetyl cysteine group(*n* = 30)	Control group(*n* = 30)	*P*-Value
Sex			
Male n(%)	22(73.4)	24(80)	0.761
Female n(%)	8(26.6)	6(20)	
Age (Year)	65.43 ± 14.850	63.00 ± 10.932	0.178
Weight (Kg)	82.23 ± 14.231	79.80 ± 12.452	0.785
LVEF%	45.67 ± 5.371	48.83 ± 4.086	0.181
Diabetes (n%)	27(90)	23(76.6)	0.381
Smoking (n%)	25(83.3)	20(66.6)	0.326
HTN (n%)	24(80)	27(90)	0.236
HLP (n%)	23(76.6)	24(80)	0.501
Preoperative Hb(g/dl)	12.3 ± 1.6	13.2 ± 2.0	0.001
Duration of surgery (min)	204.67 ± 41.646	211.00 ± 34.476	0.524
CPB Time (min)	44.33 ± 11.198	46.67 ± 9.679	0.391
ACC Time (min)	59.00 ± 14.468	64.83 ± 25.680	0.213

*LVEF* Left ventricular ejection fraction, *HTN* Hypertension, *HLP*, Hyperlipidemia, *Hb* Hemoglobin, *CPB* Cardiopulmonary bypass, *ACC* Aortic cross clamp.

Data are expressed as mean ± SD. The t-test was statistical analysis

Although serum BUN level was in the normal range in both groups, it was significantly lower in the NAC group than in the Control group at 4 h (21.25 ± 12.92 versus 28.32 ± 20.23; *P* = 0.02) and 48 h after surgery (20.03 ± 7.330 versus 25.93 ± 6.332; *P* = 0.001).

Although serum Cr level was in the normal range in both groups, it was significantly lower in the NAC group than in the Control group at 4 h (1.233 ± 0.307versus 1.337 ± 0.298; *P* < 0.001) and 48 h after surgery (1.08 ± 0.279versus 1.17 ± 0.273; P = 0.001)(Table 2).

**Table 2:** Effect of administration of N-acetyl cysteine on renal function in the two groups

Parameter	N-acetyl cysteine group(*n* = 30)	Control group(*n* = 30)	*P*-Value
BUN (mg/dL)			
Baseline	22.07 ± 6.549	18.93 ± 6.330	0.329
4 h After surgery	21.25 ± 12.92	28.32 ± 20.23	0.02 *
48 h after surgery	20.03 ± 7.330	25.93 ± 6.332	0.001 *
P-Value	0.446	< 0.001*	
Cr(mg/dL)			
Baseline	1.293 ± 0.331	1.363 ± 0.362	0.438
4 h After surgery	1.233 ± 0.307	1.337 ± 0.298	< 0.001 *
48 h after surgery	1.08 ± 0.279	1.17 ± 0.273	0.001 *
*P*-Value	0.576	< 0.05*	

*BUN*, Blood Urea Nitrogen, *Cr* Creatinine

Data are expressed as mean ± SD. The statistical test used was the t-test and repeated measurement. *P* < 0.05; t-test analysis and repeated measurement demonstrated difference between the groups with *

There were no significant differences between groups about the requirement for renal replacement therapy (*P* = 0.657).

There was no significant difference between the two groups in terms of HR (*P* > 0/05). MAP at different times (at 4 h *P* = 0.002 and 48 h after surgery *P* < 0.001) were significantly different between the two groups (Table 3).

**Table 3:** Effect of administration of N-acetyl cysteine on hemodynamic variables

Parameter	N-acetyl cysteine group(*n* = 30)	Control group(*n* = 30)	*P*-Value
HR (beat/mi)			
Baseline	78.20 ± 9.163	80.93 ± 11.608	0.685
4 h After surgery	76.07 ± 11.712	78.53 ± 10.391	0.872
48 h after surgery	75.67 ± 13.583	76.50 ± 8.842	0.896
P-Value	0.456	0.576	
MAP (mmHg)			
Baseline	119.77 ± 19.163	133.77 ± 24.241	0.324
4 h After surgery	92.87 ± 12.190	98.10 ± 22.550	0.002 *
48 h after surgery	82.20 ± 14.346	85.80 ± 13.499	< 0.001 *
P-Value	< 0.05*	< 0.05*	
U.OP (cc)			
Baseline	250.00 ± 125.945	220.33 ± 125.382	0.364
4 h After surgery	324.00 ± 124.224	306.33 ± 167.548	0.644
48 h after surgery	1161.67 ± 372.708	1032.00 ± 448.179	0.228
*P*-Value	< 0.001*	< 0.001*	

*NAC* N-acetyl cysteine, *HR* Heart rate, *MAP* Mean arterial pressure, *U.OP*: Urine out put

Data are expressed as mean ± SD. The statistical test used was the t-test and repeated measurement. *P* < 0.05; t-test analysis and repeated measurement demonstrated difference between the groups with *

Patients preserved with NAC had less bleeding in the 48-h postoperative period. Bleeding in the first 48 h was 1328 ± 895 mL in the NAC group and 1590 ± 1151 mL in the control group which was statistically significant (*P* = 0.036).

Results showed that there was a significant difference between the two groups in terms of the unit of Packed red cell transfusion (2.10 ± 1.094 versus2.80 ± 1.126; *P* = 0.002) and FFP transfusion (1.02 ± 1.548versus1.63 ± 1.377; *P* < 0.001(Table 4).

**Table 4:** Effect of administration of N-acetyl cysteine on Blood products transfusion

Parameter	N-acetyl cysteine group(*n* = 30)	Control group(*n* = 30)	*P*-Value
Postoperative bleeding in the 48 h after surgery (cc)	1328 ± 895	1590 ± 1151	0.036*
P.C	2.10 ± 1.094	2.80 ± 1.126	0.002*
FFP	1.02 ± 1.548	1.63 ± 1.377	< 0.001*

*P.C*, Packed cell, *FFP* Fresh Frozen Plasma

Data are expressed as mean ± SD. The statistical test used was the t-test. *P* < 0.05; t-test analysis demonstrated difference between the groups with*

Based on the results, the amount of urine Out Put was more in the intervention group than control at all times, but there was not significantly(*P* > 0.05)(Table 3).

## Discussion

The main purpose of this study was to investigate the effect of NAC on renal function in patients undergoing elective coronary artery bypass graft surgery.

According to the results of the study, patients of the group receiving NAChad a greater decrease in the level of Creatinine and BUN, indicating the positive effect of this drug on renal function.

In recent years, research on NAC has been on the rise due to its protective effects on organ injuries caused by oxidative stress, especially kidney function [14]. However, there are some contradictory reports about the protective effects of NAC on the kidneys. Fisher et al., have shown that N-acetyl cysteine has beneficial effects on kidney function after heart surgery, which is consistent with the results of this study [15]. However, in a study conducted by Burns on patients undergoing CABG, and in a study by Song et al., they were found that there was no difference between patients of the NAC and placebo groups in the risk of kidney function impairment [16, 17]. This may be due to some differences such as drug dosage or duration of follow-up.

Oxidative stress can be caused after reperfusion in cardiopulmonary bypass (CPB) [18]. Renal dysfunction, especially in high-risk patients, is a common problem after cardiac surgery. To control this risk factor, in addition to routine treatments, it requires a more effective medication with fewer adverse effects. Researchers have studied many antioxidant molecules and have shown that it is only some molecules (such as NAC and superoxide dismutase) that can show a positive impact on renal function and even improve it [19, 20].

In Mainra et al. study, the effect of NAC administration on renal function in patients with chronic kidney disease was evaluated. In the study, 600 mg NAC was orally prescribed for 30 patients and serum levels of Creatinine were measured at 4, 24, and 48 h. Based on the results of this study, by our findings, administration of NAC did not affect serum levels of Creatinine in these patients. Therefore, further studies with higher doses of drugs and long-term follow-up periods were suggested [21].

In the study of Rehman et al.,in the same group of patients, administration of NAC did not affect serum levels of Creatinine [22].

The effect of NAC on renal function of patients with acute and chronic renal failure was investigated by Moist et al. NAC (1200 mg) was administered at intervals of 12 h. Finally, there was no significant effect on serum creatinine levels in the intervention group compared to the placebo group [23].

The results of these studies were inconsistent with the findings of our study.

Several studies have been conducted to evaluate the protective effects of NAC on vital organs and tissues of the body in patients undergoing cardiac surgery. In a studyby Amini et al., found that treatment with NAC was effective in preventing kidney damage and its association with CABG-related deaths [24]. This is partially similar to the present study.

Şavluk et al., in a study stated that intravenous administration of NAC is associated with improved kidney function tests in patients with renal failure [25]. These findings are partly by our findings.

Aldemir et al. achieved results consistent with our study during a clinical trial to investigate the effect of NAC on performance and post-CABG renal function tests. Plasma creatinine levels in the placebo group at 24 h after surgery were significantly higher than the NAC group. In this study, they found that intravenous administration of NAC in elderly patients undergoing surgery prevents acute kidney damage and improves the results of renal function tests [26].

In a meta-analysis study in patients undergoing angiography, Song concluded that there is a significant difference in using NAC in preventing nephropathy following coronary angiography, and further studies on the use of NAC among high-risk patients are necessary [27]. In another study, Ozaidan et al. concluded that the use of NAC was effective in reducing the incidence of acute kidney damage following CABG [28]. Both of these studies are in line with the results of the present study.

Wang et al. studied the effect of NAC administration on acute kidney damage after coronary artery bypass graft surgery and concluded that serum creatinine levels were the same in both groups, but the urinary output was lower in the control group than in the NAC group. They stated that NAC administration does not prevent the progression of acute kidney damage after surgery and further studies are needed [29]. Although this study is consistent with our study in urinary output indicator, the effect of NAC on serum creatinine levels is different. The reason for this difference can be due to the variance in the sample size of the two studies.

The results of the present study showed that NAC significantly improves renal function tests and can be effective in reducing ischemic renal damage. This effect of NAC can be considered as a result of the effects of this drug on the glutathione system or due to the anti-inflammatory and antioxidant effects of NAC.

### Limitations

This study has several limitations. First; the sample size was small second; this study was single-centered. Another limitation of this study was the lack of complications in patients.

We recommended future trials with large sample size, multi-center, and long duration of follow-up.

## Conclusion

In conclusion, the administration of NAC can be reduced BUN and Creatinine levels and the incidence of kidney injury in patients undergoing CABG and mitigates the negative effect of CPB on kidney function.

## Data Availability

All data were retrieved from the institutional database and are available from the corresponding authors upon reasonable request.

## Abbreviations

CABG: coronary artery bypass graft
CPB: Cardio pulmonary bypass
AKI: Acute kidney injury
KDIGO: Kidney Disease Improving Global Outcomes
ICU: Intensive care unit
RRT: Renal replacement therapy
